# Salivary Stress/Immunological Markers in Crohn’s Disease and Ulcerative Colitis

**DOI:** 10.3390/ijms21228562

**Published:** 2020-11-13

**Authors:** Alberto Finamore, Ilaria Peluso, Omar Cauli

**Affiliations:** 1 Research Centre for Food and Nutrition, CREA (Council for Agricultural Research and Economics), Via Ardeatina 546, 00178 Rome, Italy; ilaria.peluso@crea.gov.it; 2 Department of Nursing, Faculty of Nursing and Podiatry, University of Valencia, c/Jaume Roig s/n, 46010 Valencia, Spain; 3 Frailty and cognitive impairment organized group (FROG), University of Valencia, 46010 Valencia, Spain

**Keywords:** inflammatory bowel disease, saliva, immune system, cytokines, stress, oxidative stress

## Abstract

There is continuous and growing interest in research into new alternatives to standard biomarkers to detect and follow-up disease, reducing physical and psychological stress in patients needing regular and invasive medical examinations for the evaluation of pathologies, including inflammatory bowel diseases (IBD). Saliva is one of the most promising body fluids in the research of new biomarkers, thanks to the large number of molecules it contains. Many molecules present in saliva are often directly correlated to their concentration in the blood but may be affected by the condition of the oral cavity. This means that a careful selection of a specific biomarker is required for each pathology, especially pathologies such as IBD, which may induce inflammation in the oral cavity. Here, we analyze the currently used and the proposed new salivary biomarkers (i.e., calprotectin, cytokines, IgA, cortisol, and oxidative stress markers) for the detection and follow-up of the main subtypes of IBD, known as ulcerative colitis and Crohn’s disease.

## 1. Introduction

Saliva is a very complex fluid containing a wide range of molecular components and molecules, including enzymes, hormones, antibodies, and growth factors, and it has recently been considered a potential alternative to blood- and tissue-based diagnostics for many diseases. Functionally, saliva is important for digestion, hydration of the oral mucosa, and protection of the teeth, and it is also involved in protection from oral inflammations thanks to its antimicrobial component. Mouth homeostasis and oral health (caries and periodontal disease) have been shown to largely depend on several factors, including saliva composition and its antimicrobial component and inflammatory bowel disease (IBD); patients frequently present hyposalivation [1,2] and a reduction in the antimicrobial peptides present in saliva [3]. Saliva composition and the molecular constituency of oral fluids are regulated by the transcellular or paracellular passage of molecules from the blood to saliva. These observations suggest that circulating biomarkers associated with disease may be secreted by the salivary glands, leading to changes in the biochemical composition of salivary secretions that could provide information on the individual’s health [4].

The discovery of health and pathological biomarkers in blood is well-standardized, but recent trends suggest a growing interest in finding new diagnostic biomarkers in biological specimens other than blood, such as saliva and urine. Saliva and urine sampling are highly promising due to their noninvasive natures [5]. A saliva sample collection is particularly promising, thanks to its sampling characteristics, including noninvasiveness, stress reduction, reduced risk of infection, and ease of collection and preprocessing. For instance, the advantages of using saliva are its elimination of the need for specialized technicians in collection, and the patients are not stressed, as they would be for venipuncture. There is no need for anticoagulation treatment after collection, and less manipulation than blood is required; the samples are easier to ship and store, the procedure is economical, and healthcare costs are reduced [6].

The use of saliva for diagnostic purposes has several advantages, and it is already currently used to diagnose several diseases, such as neurological diseases [7], Cushing’s syndrome [8,9], human immunodeficiency virus (HIV) [10], cancer [11], and for the follow-up of several pathologies. Several commonly used saliva biomarkers have been identified to date, and they are often comparable to the traditional measurements of inflammatory biomarkers in blood and serum. Despite these positive attributes, the use of saliva as a diagnostic fluid has yet to be investigated for several pathologies. 

Saliva contains several components, including inflammatory markers; hormones; and other molecules, including inflammatory cytokines, C reactive proteins (CRP), adipokines, insulin, cortisol, and glucose, which are currently used as markers of inflammation, cardiovascular disease, type 2 diabetes, and stress in at-risk populations [12,13,14]. However, several molecules found in saliva are found at substantially lower levels than in their concentration in blood. For instance, many of the cytokines present in salivary fluid are in inverse proportion to the salivary flow rate, while, on the contrary, CRP and cortisol are unaffected by the salivary flow rate. Salivary cortisol maintains an equilibrium with its concentration free in plasma [15], and free (unbound) cortisol present in the blood is able to cross membranes mainly by passive diffusion and, therefore, appears in several fluids, including saliva, reflecting the fact that the flow rate of the serum cortisol level is independent [16]. 

A study comparing the concentration of biomarkers in blood and saliva reported that, of the 27 cytokines analyzed in blood and saliva (collected using the passive drool method), only three cytokines detected in saliva (interleukin (IL)-6, interferon (IFN)-γ, and macrophage inflammatory protein (MIP)-1β) presented a correlation with their amount observed in blood [17], and the time it takes for a blood biomarker to pass from blood to saliva is unknown and should be established in salivary analysis studies [18].

The etiology of IBD is complex and still not fully understood, but it is assumed to be multifactorial. Indeed, IBD is characterized by a chronic intestinal inflammation, which is immune-mediated, and environmental factors, including diet, lifestyle, antibiotic use, and gut microbiota. Genetics are known to play a key role in the induction of inflammation. The prevalence of IBD is gradually increasing worldwide [19].

Ulcerative colitis (UC) is an idiopathic intestinal inflammatory disease with inflammation characteristically restricted to the mucosal surface. The disorder starts in the rectum and generally extends proximally throughout the entire colon [20].

Crohn’s disease (CD) is a chronic inflammatory disease of the entire gastrointestinal tract. It has been well-established that the mouth may be involved in the disease, with inflammatory lesions in the oral cavity [21].

The diagnosis of IBD is currently based on a combination of symptoms: biological, endoscopical, radiological and histological evaluations, and on their evolution over time [22]. Despite the exponential increase in the knowledge of the pathogenesis of IBD, an invasive diagnosis remains necessary to confirm the state of pathology and its management, and as such, new noninvasive diagnostic tools are necessary to reduce the psychological and physical stress for patients with IBD.

Interestingly, not all these studies have evaluated the impact of the presence of periodontal disease or other oral inflammations that could affect the salivary content, leading to confounding results due to altering the levels of the biomarkers in saliva [23].

In this review, we aim to discuss the principal and newly proposed inflammatory and stress biomarkers in saliva related to the pathophysiological mechanisms and symptomatology in inflammatory bowel diseases.

## 2. Calprotectin

Calprotectin is a calcium-binding complex protein constituted by two subunits. It is predominantly expressed in neutrophil and inflammatory monocytes/macrophages, although, under specific conditions, its expression has been observed in several cell types, including epithelial cells, endothelial cells, fibroblasts, keratinocytes, and osteoclasts [24]. Calprotectin is present in a large variety of fluids, such as human plasma, urine, and cerebrospinal fluid, as well as in saliva. It performs numerous biological functions, including immunoregulation, oncogenesis, and inflammation [25]; reflects the proinflammatory activities mainly induced by activated granulocytes; and is involved in leukocyte recruitment and cytokine secretion in inflammatory districts [26]. Calprotectin has also been shown to be involved in protection against pathogen infections, as indicated by experiments demonstrating that epithelial cells expressing calprotectin are more resistant to bacterial infections than epithelial cells that do not express calprotectin [27]. The monitoring of fecal calprotectin protein is common in clinical practice, and this measure is regarded as a surrogate marker for endoscopic disease activity and gives clinicians some idea of patients’ intestinal inflammatory status without performing an endoscopy [28]. In acute-phase inflammatory reactions, calprotectin is detectable in elevated amounts and is correlated to elevated levels of CRP [29]. 

A recent study has suggested saliva calprotectin as a potential index of active IBD [30]. Calprotectin concentrations in stimulated whole saliva are up to three times higher in patients with IBD compared to healthy controls, and the saliva calprotectin concentration is higher in stimulated patients with IBD compared to the controls [30]. However, while it increased in both unstimulated and stimulated patients with CD, in patients with UC, it only increased in stimulated samples. A possible explanation for these differences between CD and UC may be due to the tissue alterations present in the oral mucosa of patients with CD compared to UC [30]. Calprotectin, also known as the migration inhibitory factor—related proteins 8 and 14, is an acute-phase protein with a role in the regulation of neutrophil migration, and its concentration correlates with neutrophil migration and reflects the inflammation severity in IBD. In conclusion, calprotectin in saliva could be used as a prognostic marker, as well as an index associated with the effectiveness of therapy. However, clinicians must take into account that calprotectin secretion is also affected by oral inflammation, obesity, oral candidiasis, and periodontal disease [24,31,32].

## 3. Cytokines

The progress of research in recent years has underlined the importance of inflammatory cytokines, as it shows their fundamental role in IBD pathogenesis in terms of regulating the initiation, augmentation, and perpetuation of IBD. Cytokines are also directly involved in mucosal damages in both CD and UC [33]. The relationship between concentrations of salivary cytokines and their amount in plasma has been analyzed by Williamson et al. [17]. Twenty-seven cytokine biomarkers have been identified in the saliva of healthy adults, although only IL-6, IFN-γ, and MIP-1β showed a significant correlation with the plasma levels [17], indicating that salivary cytokine levels are affected by the oral environment and the influence of local immunity but, also, by diurnal patterns [34], although a strong correlation between saliva inflammatory biomarkers and their concentrations in the serum is largely present in healthy, as well as obese and diabetic, individuals and inflammatory conditions in children and adults [12]. Despite the action mechanisms of cytokines not being entirely clear, several studies have highlighted their involvement in the pathogenesis of IBD. 

The role of cytokines in UC pathogenesis is well-characterized [35]. An atypical T-helper (Th)2 immune response is present in UC, with high levels of the proinflammatory cytokines in addition to IL-6, IL-10, and IL-13, whereas CD4^+^ lymphocytes with the Th1 phenotype predominate in patients with CD, characterized by the production of IFN-γ and IL-2. The use of salivary cytokines as biomarkers of the inflammatory status in active and nonactive IBD is considered one of the new frontiers in the follow-up of these pathologies. As shown in Table 1, and described below, the studies conducted on salivary cytokines to date agree that IL-6 may be a marker for IBD. In a recent study on the frequencies of caries in patients with CD, IL-6 and tumor necrosis factor (TNF)-α have been observed as increased in the saliva of patients compared to controls. In the latter study, saliva was collected, and the salivary flow rate, pH, and IL-6, and TNF-α levels were examined. The salivary IL-6 and TNF-α levels in patients with CD were higher than in the controls and high in the subgroup of patients with a higher decay-missing-filled index (DMF-T). They also observed higher DMF-T and salivary flow rates in patients with CD compared to the controls, whereas the pH was lower in patients with CD than in the controls. The statistical analysis showed a positive correlation of CD duration, CRP, and IL-6 and TNF-α levels with the DMF-T. The authors therefore assumed that the increase in both IL-6 and TNF-α levels may be due to several factors, including their local secretion by the inflammatory cells of the oral mucosa and increased production by the inflammatory cells present in infected dental pulp during the caries process [36]. The increase in inflammatory cytokines in the saliva in patients with CD is consistent with a previous prospective study by Szczeklik and colleagues [21]. In this study, patients with active and nonactive diseases were enrolled and compared to healthy controls in order to evaluate whether salivary concentrations of IL-1β, IL-6, and TNF-α are associated with the activity and oral manifestations of CD, such as oral lesions. The results showed that oral lesions characteristic of CD were present in patients with the active disease, whereas nonspecific oral lesions were present in the groups of both active and nonactive CD. The cytokine analysis in saliva revealed a significant increase in the levels of IL-1β, IL-6, and TNF-α in active CD compared with inactive CD and the controls, which was correlated with the presence of characteristic oral lesions. Higher levels of these proinflammatory cytokines were also correlated with clinical and biochemical markers of the disease activity [21]. The results for increasing the IL-6 concentration in saliva confirmed the previous observations by Nielsen and colleagues [37], who found that IL-6 was higher in the saliva of both patients with UC and CD than in the control subjects. They also showed that the plasma IL-6 of patients with CD correlated significantly with CRP and albumin, as well as in UC, where salivary IL-6 was found to be correlated with the IL-6 serum concentration. They concluded that the concentration of IL-6 is directly involved with the gastrointestinal tract and the mouth cavity, and the IL-6 salivary concentration may therefore be considered an additional method for evaluating and monitoring the disease’s activity [37]. Another study conducted only on patients affected by UC (16 newly diagnosed and 16 treated with medication) and 16 healthy controls evaluated the amount of salivary IL-6, CRP, and albumin. All the patients were selected as having the proctosigmoiditis type of UC, and in the medication group, all patients were being treated with sulfasalazine as the anti-inflammatory drug. IL-6, CRP, and albumin were analyzed in unstimulated saliva. All markers were increased in the newly diagnosed UC patients compared to the healthy controls. In the UC patients treated with the anti-inflammatory drug, the salivary marker levels were lower than the newly diagnosed UC patients, although they were higher than the control subjects. Moreover, there is a relation between the oral findings observed in UC patients and the salivary IL-6, CRP, and albumin levels. They also concluded that IL-6 is an indicator of an inflammatory process in the bowel, since saliva-producing cells are considered part of the digestive tract. The increase in the level of CRP is not surprising, because it is a sensitive index of the inflammatory status [38]. 

Among the several cytokines involved in the pathogenesis of UC, transforming growth factor (TGF)-β1 seems to play a key role. Compared to healthy subjects, it has been observed to increase gene encoding for TGF-β1 in tissue from patients with active and nonactive UC, and the serum concentration of TGF-β1 was significantly higher in patients with the active disease [39]. A very recent study has also reported that methylation in the promoter of the TGFβ1 gene has a high discriminative power for distinguishing CD from UC and could be useful as an important diagnostic marker [40]. TGF-β1 and reactive nitrogen species such as nitric oxide (NO) have been also analyzed in the saliva of patients affected by UC. The authors hypothesized that different levels of NO and TGF-β1 in saliva might change on the basis of the UC severity, and their levels might be useful for determining the activity of the disease. Thirtyseven UC patients with different degrees of severity (8 severe, 21 mild, and 8 moderate) and 15 healthy controls were enrolled in this study, and TGF-β1 and NO were detected in the saliva and compared between the groups. The results indicated significantly higher levels of NO and TGF-β1 in the UC patients compared to the healthy subjects, although no differences were observed among the UC group. They therefore concluded that, despite the increase in NO and TGF-β1 in the UC patients, these markers cannot be useful in predicting the activity of the disease [41]. In a previous study, the same authors also investigated the levels of TGF-β1 and NO in saliva, as well as the salivary levels of the total antioxidant capacity; specific antioxidant molecules such as uric acid, albumin, transferrin, and thiol; and lipid peroxidation in patients with CD and control subjects in order to investigate their correlation with the activity of the disease. Patients with different levels of severity and healthy controls were included in the study. The results showed that there was a significant reduction of salivary levels of the total antioxidant capacity-specific antioxidants in CD, whereas the lipid peroxidation increased. The authors also observed that the lipid peroxidation and antioxidant total capacity were significantly correlated with the Crohn’s disease activity index (CDAI). TGF-β1 and NO were significantly higher in patients with CD compared to the healthy subjects. Based on the results observed, they concluded that oxidative stress may affect the determination of other markers. They also underline the importance of oxidative stress in the pathogenesis of CD [42]. A significant increase in the epidermal growth factor was also observed in the saliva of patients with CD [43].

In a very interesting study conducted by Majster and colleagues [44], ninety-two inflammation-related proteins were analyzed in the serum and saliva of patients affected by IBD. Twenty-one patients with CD and UC and healthy controls were recruited for this study. Plasma and unstimulated and stimulated saliva were collected at the start, and all patients with IBD were invited for saliva and plasma resampling after treatment. The authors analyzed the salivary and circulatory inflammatory profiles of patients with IBD with active intestinal inflammation before and after drug treatment, and they also explored the link between their expression and activity of IBD. They observed that most of the proteins analyzed were detected in the saliva and in plasma but with a different pattern of expression in the two fluids. In support of the hypothesis that salivary proteins may be analogous to circulatory biomarkers, they observed that most of the inflammatory proteins analyzed were found in the serum and in both unstimulated and stimulated saliva. Patients with IBD presented significantly higher levels of IL-6 and metalloproteinase (MMP)-10 in stimulated saliva, and the authors concluded that IL-6 and MMP-10 seemed accurate in distinguishing patients with IBD from healthy controls. IL-6 and MMP-10 are known to be involved in the pathogenesis of IBD, as shown by previous studies that indicated that they are elevated in the serum and in the inflamed and in nonlesional intestinal mucosa of patients with IBD [45,46]. MMP-10 was also significantly correlated with the CD score. In conclusion, the results observed suggested that IL-6 and MMP-10 in the stimulated saliva were associated with the pathogenesis and extraintestinal manifestations of IBD, indicating that saliva could provide noninvasive disease markers for IBD. 

In conclusion, as regards the use of cytokines as biomarkers of IBD, despite the many studies conducted, IL-6 seems to be the most promising biomarker. However, the salivary cytokine concentration is affected by several factors, including inflammation of the oral cavity, active phase of the disease, oral microbiota composition, salivary flow rate, and periodontal disease for both CD and UC. 

## 4. Exosomes

Exosomes contain several components, including nucleic acids, several proteins, and lipids and chemical drugs. Exosomes may transport all these molecules from inflammatory sites to other tissues to facilitate or alleviate pathological processes [48,49], and they are involved in the immunological processes such as modulating T and B-cell differentiation, activation and differentiation, and modulating innate immune responses. Exosomes have also been suggested as a new therapeutic strategy to reduce inflammation associated with IBD, hence the recent idea to use exosomes in the diagnosis and therapy of IBD [50,51]. 

In comparison to exosomes from the colonic lumen of healthy subjects, exosomes isolated from patients with IBD have been shown to contain higher amounts of IL-6, IL-8, IL-10, and TNF-α. Moreover, the levels of these proinflammatory cytokines were positively correlated with the CD severity score, and in vitro experiments indicate that exosomes from patients with IBD induce the activation of colonic epithelial cells in vitro and in IL-8 secretion [51]. Exosomes can be released in all biological fluids, and an analysis of the changes in the exosomal content may therefore be considered as a resource in the identification of noninvasive biomarkers for infectious pathologies and IBD [52]. Despite the extensive literature on the role and presence of exosomes in tissue and biological fluids in several pathologies and infections, only Zheng X. and colleagues [53] have investigated the presence of exosomes in IBD patients with saliva. The author suggested that the saliva of patients with IBD contains exosomes, and its contents may be useful as biomarkers of IBD due to the characteristics of exosomes. The authors used a proteomic approach to identify the protein composition of exosomes in the saliva of patients with IBD and healthy subjects and to examine the differences in protein compositions. They recruited 48 patients with IBD (37 had UC and 11 CD) and 10 healthy subjects without IBD as controls. They observed major differences in the protein profiles of the three groups. In particular, they observed that eight proteins were only expressed in the exosomes of patients affected by IBD, and these observed proteins were involved in inflammation and the immune response. The authors showed that, in comparison to healthy subjects, the proteasome subunit alpha type 7 (PSMA7) expression level was higher in patients with IBD, suggesting PSMA7 is a good biomarker for IBD diagnosis.

## 5. Amylase and Mucin 5B

Amylase is an enzyme produced primarily by the pancreas, and it is also secreted by the parotid glands (salivary alpha amylase: sAA) and is necessary for carbohydrate digestion. Mucins are mainly secreted in saliva by the submandibular and sublingual glands, and it is a glycoprotein able to confer the viscoelasticity in saliva [54]. A study on CD activity conducted on 53 patients with active or nonactive diseases found that the disease activity did not correlate with the saliva flow rate, the concentration of amylase and amylase output, the Mucin 5B (MUC5B) concentration, or the ratio of amylase:MUC5B. However, the authors observed a statistically significant correlation with the MUC5B output. In this study, the authors concluded that the salivary flow did not correlate with xerostomia complaints, indicating that dry mouth complaints perceived by patients with IBD were not necessarily associated with a reduction in saliva secretion rates. Despite the lack of change in the salivary flow rate, the composition of saliva might change during the active disease, as indicated by the increased mucin 5B output [2]. By contrast, a more recent study conducted on patients with UC [1] reported no correlation between disease activity and MUC5B. The authors did not observe a link between the parameters analyzed (salivary amylase activity, MUC5B concentrations, or the ratios of amylase:MUC5B) in either stimulated or unstimulated saliva or in the xerostomia inventory score or intestinal disease activity. However, a correlation between the quality of life in patients with IBD and the xerostomia inventory score with the simple clinical colitis activity index was reported [1]. Meanwhile, an increase in the concentration of alpha-amylase compared to healthy controls was observed in both the stimulated and unstimulated saliva of UC patients, although no significant changes were observed in the flow rates. Changes in alpha-amylase secretion were not associated with an increase in the number of copies of the gene responsible for alpha-amylase [55]. The authors of the latter study concluded that the elevated alpha-amylase secretion in UC patients might be due to the involvement of sympathetic overactivity in the UC population. Despite the reasonable use of salivary alpha-amylase as an inflammatory biomarker of IBD, changes in the saliva alpha-amylase levels may be due to psychological stress. Indeed, alpha-amylase is an enzyme that increases with acute stress and is correlated to sympathetic nervous system (SNS) neurotransmitter release [56]. Moreover, normal baseline sAA levels display diurnal patterns and awakening responses, which should be taken into account during saliva sampling [57,58].

## 6. Salivary IgA

Secretory Immunoglobulin A (SIgA) is the main immunoglobulin found in salivary glands, which play a key role in protecting from infection vulnerable tissues such as the oral cavity, lungs, and gut [59]. SIgA in saliva interacts with the autonomic nervous system, changing its concentration in response to physical and psychological stresses [60,61,62] and changing the saliva flow rate [63]. SIgA antibodies are present in saliva and are able to react to a large variety of indigenous bacteria. These antibodies play a fundamental role in controlling oral microbiota due to their capacity to reduce the bacteria’s adhesion to the oral mucosa and teeth [64]. The microbiota is known to play a key role in the health and diseases of both humans and animals, and several studies have attempted to understand the role of oral microbiota in health and disease and regulate the secretion of immunological markers. Since oral lesions commonly appear in patients with CD rather than in healthy subjects or patients with UC, it has been suggested that a reduction in local IgA production favors the bacterial invasion of oral mucous, leading to the production of oral lesions. Crama-Bobbouth et al. [65] reported that, in comparison to healthy controls, patients with CD showed a significant increase of IgA, IgM, and IgG levels in saliva, and no correlation with the activity of the disease was present.

The increased frequency of oral manifestations among patients with IBD has recently been shown to be associated with several changes in the oral microbiota, as indicated by the study by Heba Said et al. [47], which observed significantly increased *Bacteroidetes* associated with a reduction in *Proteobacteria* in the salivary microbiota of patients with IBD. The dominant genera found in the saliva of patients with IBD were *Streptococcus*, *Prevotella*, *Neisseria*, *Haemophilus*, *Veillonella*, and *Gemella*, which were the cause of the dysbiosis observed in the salivary microbiota of those patients. These results are consistent with previous studies, which showed how oral microbiota occur after periodontal therapy and/or periodontal infection [66,67]. Heba S. Said and colleagues enrolled patients with IBD (14 CD and 10 UC) and 15 healthy controls and, also, performed an analysis of immunological biomarkers in the saliva of IBD, such as cytokines, secretory IgA, and lysozymes. The results indicated that lysozyme levels were lower in the saliva of both the CD and UC groups compared to the healthy controls, whereas the levels of IgA and cathelicidin antimicrobial peptides (CAMP) LL-37 in both the CD and UC groups were significantly higher than the controls. IL-1β was significantly higher in both CD and UC compared to the controls, while levels of IL-6, IL-8, and monocyte chemoattractant protein (MCP)-1 resulted significantly higher only in the saliva of the patients with UC, while higher levels of TNF-α were found in patients with CD. The levels of IgA and MCP-1 were significantly higher in the saliva of patients with UC than those in the CD group, indicating that oral cavity patients with IBD usually result in an inflammatory state. Interestingly, they found a relationship between salivary microbiota and immunological markers, as shown by the strong correlation observed between lysozymes, the amount of IL-1β, and the relative abundance of genera such as *Streptococcus*, *Prevotella*, *Haemophilus*, and *Veillonella*. The analysis of salivary IgA should be performed considering the oral health of patients other than inflammatory intestinal disease, and salivary IgA are largely affected by oral microbiota and teeth health, and dental caries are associated with an increase in salivary IgA that appear to be involved in dental caries control [64,65,66,67,68,69]. 

While many studies have characterized the salivary secretory level of IgA in patients with CD, only a few have been performed on patients with UC, providing contradictory and not exhaustive results. In these studies, the authors concluded that the salivary IgA concentrations in UC patients did not differ from healthy subjects [70,71]. In conclusion, despite the analysis suggesting salivary IgA results as promising, further studies on its possible role in oral health problems in patients with UC should be performed to confirm the possibility of using IgA as an IBD marker. 

## 7. Cortisol

Increasing evidence suggests that there is a close association between IBD and stress. Stress induces the perturbation of homeostasis by affecting functions of the gastrointestinal tract, leading to the development of a broad array of gastrointestinal disorders, including IBD, due to alterations in the brain-gut interactions associated with an increase of intestinal permeability and microbiota perturbation [72]. 

It is well-known that psychological stress activates a physiological response, which is complex and regulated by the hypothalamic-pituitary-adrenal (HPA) axis and SNS [73], leading to cortisol release by the adrenal cortex and its diffusion in all tissues by blood, which seems to be associated with an increase in intestinal permeability [74]. Stress is usually assessed by using validated self-report questionnaires, which quantify the individual’s personal and subjective experience of stress. Salivary cortisol and sAA are currently used for stress research and quantification, and their increased popularity is due to their noninvasive sampling and methods and ease of quantification. Moreover, their levels in saliva are correlated to their amounts in blood [75,76].

As mentioned above, the saliva cortisol levels are not affected by the salivary flow rate, and its level is closely correlated with the blood cortisol concentrations, as well as with HPA axis activity [15], suggesting that salivary cortisol could be a marker of stress. In addition, an association between cortisol in saliva and markers of inflammation, including IL-6, IL-10, and TNF-α in plasma, has been demonstrated by the Multi-Ethnic Study of Atherosclerosis [77].

Despite stress being associated with the course of IBD and increasing during the exacerbation of symptoms, contributing to the aggravation of the symptomatology in a study conducted on pregnant women with well-controlled IBD (six with CD and seven with UC), no changes in salivary cortisol were observed in comparison to healthy pregnant women [78]. The inverse relationship between vagal tone quantified by the power spectral analysis of the heart rate variability and evening salivary cortisol level is absent in patients with UC [79]. These results suggest that there is a decoupling between the hypothalamic-pituitary axis and the autonomic nervous system in CD and suggests an alteration in the neuronal circuits between the prefrontal cortex and the amygdala, as recently shown in depression and anxiety, which could therefore be related to the high prevalence of depression and anxiety in IBD. The role of cortisol in the pathogenesis and exacerbation of IBD warrants future studies in order to assess whether it is also linked to other alterations in addition to being a suitable marker of stress and related depression and anxiety.

## 8. Salivary Oxidative Stress Markers in IBD

Oxidative stress markers include enzymes, including those that produce ROS and those that neutralize ROS, nonenzymatic antioxidants, and markers based on ROS-induced modifications [80]. Of these markers, the following have been in saliva samples of patients with IBD: enzymes, including glutathione peroxidase (GPX), superoxide dismutase (SOD), and myeloperoxidase (MPO); nonenzymatic antioxidants, such as the total antioxidant capacity (TAC), uric acid (UA), and total thiol level (SH); thio-barbituric acid reactive substances (TBARS) and ROS-induced proteins modifications, including advanced oxidation protein products (AOPP); and advanced glycation end products (AGE) (Table 2) [42,43,81,82,83,84].

The TBARS level, a marker of lipid oxidation measured with the colorimetric assay, has sometimes been improperly named malondialdehyde, which should be detected using high-performance liquid chromatography (HPLC) methods [80]. Similarly, reduced glutathione should be measured by HPCL methods [80], whereas the 5,5′-dithiobis 2 nitro-benzoic acid (DTNB)-based assay [42] evaluated SH. We used the abbreviations TBARS and SH in Table 2, which provides a concise description of the results of the case-control studies [42,43,81,82,83,84].

Two [81,82] out of six studies collected stimulated (STIM) saliva samples (Table 2), although various markers of oxidative stress were altered after stimulation compared to unstimulated (UNST) samples [85]. Furthermore, calprotectin was significantly elevated in UNST salivary samples of CD patients compared to healthy controls, but not in individuals with UC, whereas it was significantly elevated in both UC and CD patients in STIM samples, suggesting that salivary calprotectin from the UNST collection might be particularly specific for CD [30]. Moreover, patients with CD tended to have higher concentrations of calprotectin in STIM saliva, compared to patients with UC, and those with ileal CD tended to have elevated calprotectin concentrations in UNST and STIM saliva, compared to those with ileocolic and colonic CD, whereas no differences were observed within the UC group as regards the extension of the disease [30]. In addition, the levels of calprotectin in UNST and STIM saliva were 4.0-fold versus 1.4-fold higher at the baseline, respectively, in naïve patients with CD and significantly reduced in UNST saliva, whereas, in UC, the concentrations were comparable in treatment-naïve and previously treated patients [30].

Only two studies evaluated the oxidative stress markers in UC patients [43,81] using STIM and UNST salivary samples. They found conflicting results when comparing the TAC, measured in terms of ferric-reducing antioxidant power (FRAP) (Table 2). Compared to the controls, the levels of salivary TBARS were higher and FRAP was lower in patients with CD but not in patients with UC [43]. Reduced UA and TAC and increased TBARS were found in patients with CD compared to healthy individuals [42].

Moreover, in patients with CD, a negative correlation was observed between salivary FRAP and serum CRP [81], and the latter correlated with TBARS [83]. Reduced TAC and/or increased TBARS were associated with the CDAI [42,83], whereas the salivary calprotectin concentrations were not correlated with the serum calprotectin, fecal calprotectin, and CRP [30].

Majster et al. [30] suggested that subclinical immune alterations of the oral mucosa, including orofacial granulomatosis (OFG), could be reflected in the saliva. Jansakova et al. [84] recently compared individuals with OFG, patients with CD with and without OFG, and healthy controls. No differences were found between the TBARS and TAC groups measured with the 2,2′-azino-bis(3-ethylbenzothiazoline-6-sulphonic acid (ABTS) method [84]. On the contrary, FRAP was lower in all patients with CD and in individuals with OFG, whereas AGE were higher in CD and OFG + CD patients [84]. Surprisingly, AOPP values were higher in both CD and OFG groups but not in CD patients with OFG. However, unlike the other studies listed in Table 2, smokers were included in this study, and this could bias the results [84]. Moreover, all the patients with CD were in remission, and no significant differences were found in MPO compared to the healthy controls [84]. In another study, no significant differences were found compared to healthy controls in SOD or GPX activity, regardless of the CDAI [82]. The latter correlated inversely with SOD in plasma, whereas no correlations were observed between either SOD or GPX activity in saliva and the CDAI [82].

In overall terms, the data on biomarkers of oxidative stress in IBD are promising, but more studies are needed in order to better define their clinical relevance and a cut-off value for each marker.

## 9. Conclusions

The oral cavity is a complicated structure, composed of several different tissues and structures that coexist and work together to function correctly. The oral cavity is colonized by a wide variety of bacteria strains, which play an important role in homeostasis. Saliva covers all areas of the oral cavity, and it plays an important role in the protection of oral mucosa (i.e., lubrication, buffering, and protection against microorganisms) and the teeth (i.e., remineralization and protection against demineralization), as well as being an essential component of the digestive process. Saliva is constituted principally by secretions of the major and minor salivary glands, where many different components are dissolved, including mucosal transudations; serum; and blood derivatives from oral wounds, bacteria, and bacterial products, as well as hormones, proteins, enzymes, antibodies, antimicrobial constituents, and cytokines [86]. The molecular and microbial analytes present in saliva may be affected by several local and systemic disorders, and as such, salivary components may be effective as markers for both local and systemic disorders and can be good discriminatory biomarkers for local, systemic, and infectious disorders [4,87]. Several studies have been performed in order to individuate specific biomarkers for IBD. The studies performed to date have shown that saliva contains several potential biomarkers for the detection of pathologies and evaluation of their status. As described above, the cytokines present in saliva are the most extensively analyzed marker, although other analytes such as calprotectin, exosome-containing molecules, IgA, cortisol, amylase, and oxidative stress indicators have been proposed as indicators of the pathology status. Of the salivary components analyzed, IL-6 has the most interleukins studied, and the results are in accordance with an increase in its amount in the saliva both [21,36,37,38,47] in patients with CD and UC, whereas contradictory results have been observed for salivary TNF-α [21,36], circulating IL-6, and TNF-α, which are commonly used as indicators of disease activity in IBD and the state of endoscopic disease activity in IBD [88,89]. The key role of IL-6 in IBD is highlighted by the fact that the serum concentrations are directly correlated to the inflammatory disease activity. Furthermore, the inflammatory actions of IL-6 have been largely demonstrated to be associated with intestinal inflammation induction in IBD [90]. The presence of inflammatory cytokines in saliva has been strongly correlated to IBD [21,36,37], but their concentrations may be also influenced by the state of the oral cavity, as oral inflammation and periodontitis are very common in patients with IBD [23,91]. In fact, higher frequencies of oral manifestations in patients with active IBD compared to controls and those with a nonactive disease have been assumed to be a possible result of altered cytokine activity in the gastrointestinal tract, including the oral cavity. Despite the high degree of correlation between salivary inflammatory cytokines and IBD, healthy subjects affected by periodontitis have also shown a pattern of a high expression of inflammatory cytokines in gingival crevicular fluid similar to both CD and UC patients [92]. These findings suggest that a proper evaluation of the oral cavity should be performed before using cytokines as an IBD biomarker, considering also the high frequencies of oral manifestation associated with IBD. In support of this conclusion, a very recent study has shown that IL-6 is correlated to an active state of dental caries and associated with infectious consequences [93]. In addition, the authors of the latter study did not observe any significant differences in SIgA between the saliva of children with active caries in comparison to the caries-free group. An analysis of oxidative biomarkers in saliva has also shown that subjects with periodontitis and/or dental caries presented high levels of oxidative stress markers, proteomic inflammatory markers, and bacterial dysbiosis [94]. In mouse models of periodontitis, Kitamoto et al. [95] reported oral inflammatory Th17 cells and oral pathobiontic bacterial species able to colonize and translocate in the intestine, causing IBD by highlighting the gut-oral connection in IBD through dysregulated inflammatory responses originating in the oral cavity and migrating systemically. A single salivary biomarker for IBD has not yet been identified. Considering the high complexity of the structure and tissues present in the oral cavity, as well as the oral pathologies associated with IBD, clinicians should consider more than one salivary biomarker for the early diagnosis of CD or CU. It is also necessary to consider biomarkers that cannot be influenced by the local inflammatory state of the oral cavity, which could influence the release of cytokines, modifying the composition of the oral microbiota and the oxidative state of the oral cavity. In conclusion, further studies are necessary to validate all these findings and to discover salivary biomarkers that are reliable for the early diagnosis and monitoring of IBD.

## Figures and Tables

**Table 1:** Cytokine profile in the saliva of patients with inflammatory bowel disease (IBD).

IBD Severity	Control	Saliva	Salivary Biomarkers	Results	Ref
CD (*n* = 48)	Healthy (*n* = 48)	UNST	IL-6	↑	[36]
			TNF-α	↓	
			pH	↔	
			salivary flow rate	↓	
CD (*n* = 52) CDAI 256.5 ± 36.9	Healthy (*n* = 43)	UNST	IL-1β, IL-6 and TNF-α	↑	[21]
			Oral lesion	Correlated to cytokines increase	
CD (*n* = 43) CDAI 107.5 ± 30.2	Healthy (*n* = 43)	UNST	IL-1β, IL-6 and TNF-α	↔	[21]
CD (*n* = 15)	Healthy (*n* = 19)	UNST	IL-6	↑ (CD>UC)	[37]
UC (*n* = 7)	Healthy (*n* = 19)	UNST	IL-6	↑	[37]
UC (*n* = 16) newly diagnosed	Healthy (*n* = 16)	UNST	IL-6, CRP and albumin	↑	[38]
UC (*n* = 16) in medication	Healthy (*n* = 16)	UNST	IL-6	↔	[38]
			CRP and albumin	↑	
UC (*n* = 37)	Healthy (*n* = 15)	UNST	TGF-β1 and NO	↑	[41]
CD (*n* = 28) CDAI 102.1 ± 84.9	Healthy (*n* = 20)	UNST	TGF-β1 and NO	↑	[42]
CD + UC (*n* = 12 + 9)	Healthy (*n* = 22)	UNST STIM	IL-6 and MMP-10	↑ (in STIM saliva)	[44]
CD (*n* = 14)	Healthy (*n* = 15)	UNST	Secretory IgA	↑ (UC>CD)	[47]
			Lisozima	↓	
			LL37, IL-1β and TNF-α	↑	
UC (*n* = 10)	Healthy (*n* = 15)	UNST	Secretory IgA	↑ (UC>CD)	[47]
			Lisozima	↓	
			LL37, IL-1β, IL-6, IL-8	↑	
			MCP-1	↑	

CD: Crohn’s disease, CDAI: Crohn’s disease activity index, UC: ulcerative colitis, UNST: unstimulated (collection), STIM: stimulated (collection), IL-: interleukin, TNF-α: tumor necrosis factor-α, MCP-1: monocyte chemoattractant protein-1, CRP: C-reactive protein, TGF-β1: transforming growth factor-β, NO: nitric oxide, and LL37: antimicrobial peptide. ↑: increased; ↓: decreased; ↔: unchanged; >: increased compared with.

**Table 2:** Oxidative stress markers in case-control studies.

IBD Severity	Control	Saliva	Enzymes	Non-Enzymatic Antioxidants	Lipid Oxidation	Protein Oxidation	Ref.
CD (*n* = 18)	Healthy (*n* = 5)	STIM		TAC (FRAP) ↓			[81]
UC (*n* = 13)	Healthy (*n* = 5)	STIM		TAC (FRAP) ↓			[81]
CD(*n* = 25) CDAI: 262.5 ± 57.7	Healthy (*n* = 25)	STIM	GPX ↔ SOD ↔				[82]
CD (*n* = 22) CDAI: 67.2 ± 40.2	Healthy (*n* = 25)	STIM	GPX ↔ SOD ↔				[82]
CD (*n* = 16)	Healthy (*n* = 16)	UNST		TAC (FRAP) ↓	TBARS ↑		[43]
UC (*n* = 16)	Healthy (*n* = 16)	UNST		TAC (FRAP) ↔	TBARS ↔		[43]
CD (*n* = 32) CDAI: 270.8 ± 31.2	Healthy (*n* = 26)	UNST		TAC (FRAP) ↔ SH (DTNB) ↓	TBARS ↑		[83]
CD (*n* = 26) CDAI: 67.2 ± 21.4	Healthy (*n* = 26)	UNST		TAC (FRAP) ↔ SH (DTNB) ↔	TBARS ↔		[83]
CD (*n* = 28) CDAI: 102.1 ± 84.9	Healthy (*n* = 20)	UNST		TAC (FRAP) ↓ UA ↓ SH (DTNB) ↔	TBARS ↑		[42]
CD (*n* = 29) remission	Healthy (*n* = 22)	UNST	MPO ↔	TAC (ABTS) ↔ TAC (FRAP) ↓	TBARS ↔	AOPP ↑ AGE ↑	[84]
CD remission + OFG (*n* = 14)	OFG (*n* = 27)	UNST	MPO ↔	TAC (ABTS) ↔ TAC (FRAP) ↓	TBARS ↔	AOPP ↔ AGE ↑	[84]

CD: Crohn’s disease, CDAI: Crohn’s disease activity index, UC: ulcerative colitis, OFG: orofacial granulomatosis, UNST: unstimulated (collection), STIM: stimulated (collection), GPX: glutathione peroxidase, SOD: superoxide dismutase, MPO: myeloperoxidase, TAC: total antioxidant capacity, ABTS: 2,2′-azino-bis(3-ethylbenzothiazoline-6-sulphonic acid, FRAP: ferric-reducing antioxidant power, UA: uric acid, SH: total thiol level (DTNB: 5,5′-dithiobis 2 nitrobenzoic acid), TBARS: thio-barbituric acid reactive substances, AOPP: advanced oxidation protein products, and AGE: advanced glycation end products. ↑: increased; ↓: decreased; ↔: unchanged; >: increased compared with.

## Abbreviations

ABTS	2,2′-azino-bis 3-ethylbenzothiazoline-6-sulphonic acid
AGE	advanced glycation end products
AOPP	advanced oxidation protein products
CAMP	cathelicidin antimicrobial peptides
CD	Crohn’s disease
CDAI	Crohn’s Disease Activity Index
CRP	C-reactive Protein
DMF-T	decay-missing-filled index
DTNB	5,5′-dithiobis 2 nitrobenzoic acid
FRAP	ferric reducing antioxidant power
GPX	glutathione peroxidase
HIV	Human Immunodeficiency Virus
HPA	hypothalamic-pituitary-adrenal
HPLC	high-performance liquid chromatography
IBD	inflammatory bowel disease
IFN-γ	interferon-gamma
IgA	immunoglobulin A
IL	interleukin
MCP	monocyte chemoattractant protein
MIP-1β	macrophage inflammatory protein-1beta
MMP-10	metalloproteinase-10
MPO	myeloperoxidase
MUC5B	mucin 5B
NO	nitric oxide
OFG	orofacial granulomatosis
PSMA7	proteasome subunit alpha type 7
ROS	reactive oxygen species
sAA	salivary alpha amylase
SIgA	secretory immunoglobulin A
SH	total thiol level
SOD	superoxide dismutase
SNS	sympathetic nervous system
STIM	stimulated
TAC	total antioxidant capacity
TBARS	thiobarbituric acid reactive substances
TGF-β	transforming growth factor-beta
Th	T helper
TNF-α	tumor necrosis factor-alpha
UA	uric acid
UC	ulcerative colitis
UNST	unstimulated
